# Diagnosis and Management of Keppen-Lubinsky Syndrome in a Lebanese Infant: A Case Report

**DOI:** 10.7759/cureus.98872

**Published:** 2025-12-10

**Authors:** Hadi Fakih

**Affiliations:** 1 Pediatrics, Faculty of Medical Sciences, Lebanese University, Beirut, LBN

**Keywords:** dysmorphology, epilepsy, hypotonia, keppen-lubinsky, whole exome sequencing (wes)

## Abstract

We report a five-month-old male infant of Lebanese descent who presented with infantile spasms, axial hypotonia, and a distinctive facial gestalt. Whole exome sequencing (WES) identified a heterozygous pathogenic variant (c.460G>A; p.Gly154Ser) in the *KCNJ6* gene, confirming a diagnosis of Keppen-Lubinsky Syndrome (KPLBS). This case underscores the utility of WES in diagnosing rare dysmorphic syndromes, discusses the therapeutic challenges of channelopathy-related epilepsy, and outlines the guarded prognosis associated with KPLBS.

## Introduction

Keppen-Lubinsky Syndrome (KPLBS; OMIM #614098) is a severe neurodevelopmental disorder first delineated in 2015, resulting from heterozygous mutations in the *KCNJ6* gene [1]. This gene encodes the G-protein-coupled inwardly rectifying potassium channel 2 (GIRK2), which is critical for stabilizing the neuronal resting membrane potential and modulating neurotransmitter action in the brain and heart [1,2]. The dysregulation of this channel leads to neuronal hyperexcitability, which underpins the core neurological features of the syndrome. The classic phenotype, as described in the initial cohort and subsequent limited case reports (fewer than 20 reported worldwide), includes profound developmental delay, hyperreflexia, and a distinctive facial appearance often described as "aged," characterized by microcephaly, prominent eyes, a narrow nasal bridge, and a persistently open mouth [1,3]. Seizures are a common and often treatment-resistant feature, contributing significantly to the disease burden [3,4]. Due to its extreme rarity, the clinical spectrum and long-term outcomes of KPLBS are still being defined. Early recognition and genetic evaluation are crucial for definitive diagnosis, accurate prognostication, and guiding family counseling. We present a case of a Lebanese infant with a confirmed *KCNJ6* mutation, highlighting the clinical journey, expanding on the management challenges of this specific channelopathy, and discussing the prognostic implications in the context of existing literature.

## Case presentation

A five-month-old male infant, the second child of non-consanguineous Lebanese parents with a healthy first-born child, was admitted to our pediatric department for evaluation of recurrent paroxysmal events. The mother reported episodes described as “eye rolling,” drooling, and infantile spasms, leading to multiple emergency room visits. The perinatal history was unremarkable, with no birth asphyxia or NICU admission. There was no family history of neurological or epileptic disorders.

**Figure 1:**
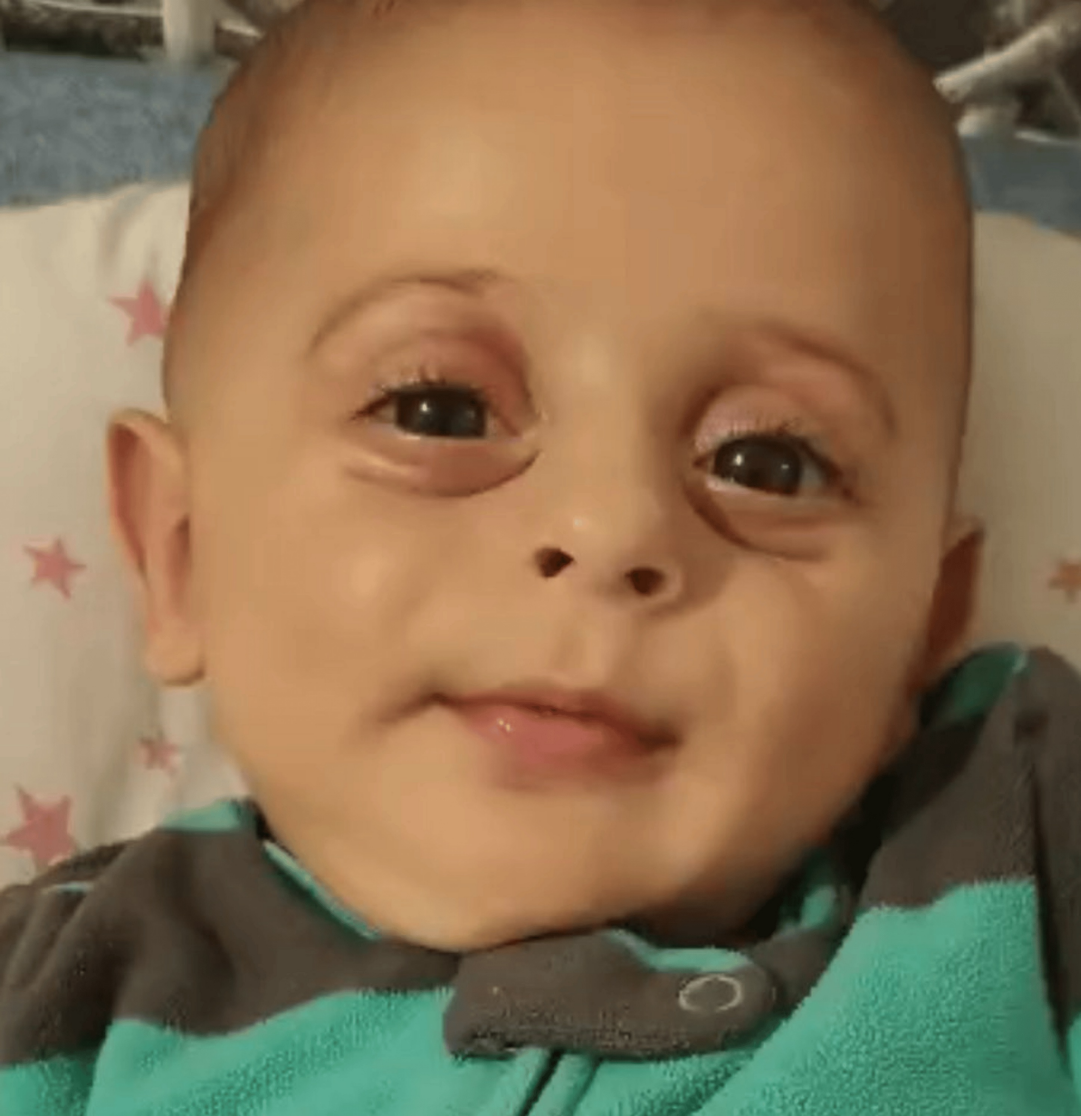
Facial photograph of the patient showing prominent eyes, infraorbital wrinkles, long philtrum, and broad nasal bridge. Written informed consent for the publication of the patient's photograph was obtained from the patient's guardian, and a signed form has been submitted to the journal.

Physical examination revealed significant axial hypotonia with a severe head lag. Cranial and peripheral nerve examinations were normal. Distinctive dysmorphic features were noted (Figure 1), including protruding eye globes, marked infraorbital skin wrinkling, a long philtrum, a broad nasal bridge, and a broad forehead.

**Figure 2:**
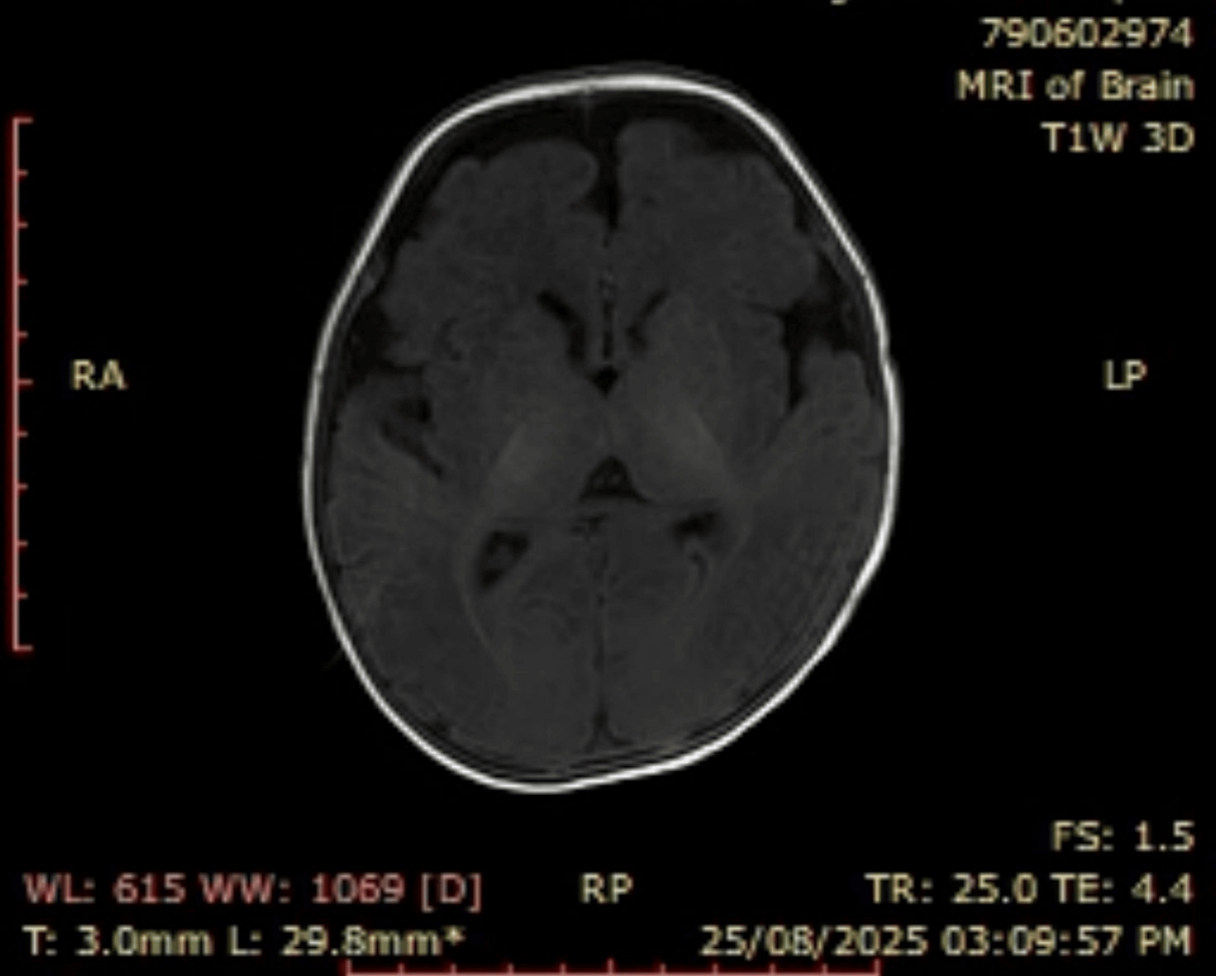
Axial T1-weighted MRI image of the brain, demonstrating normal parenchyma and myelination for age, with no structural abnormalities. MRI: magnetic resonance imaging

Anthropometric measurements placed his weight and height at the 10th percentile, while his head circumference was normal, ruling out microcephaly. Initial workup showed mild lactic acidosis and an elevated creatine phosphokinase (CPK) level. Brain magnetic resonance imaging (MRI) was interpreted as normal for age (Figure 2). The electroencephalogram (EEG) was normal despite the clinical seizures. A fundoscopic examination was unremarkable. Given the constellation of neurological symptoms, dysmorphism, and inconclusive routine investigations, clinical whole exome sequencing (WES) was pursued [5].

WES identified a heterozygous pathogenic variant (NM_002240.5:c.460G>A; p.Gly154Ser) in the KCNJ6 gene, consistent with a diagnosis of KPLBS [1], with no relevant copy number variants detected. The variant was classified as pathogenic based on the American College of Medical Genetics and Genomics (ACMG) criteria [2].

## Discussion

This report describes a classic presentation of KPLBS, confirmed molecularly in a Lebanese infant, thereby contributing to the geographic and ethnic diversity of reported cases. The patient’s phenotype aligns closely with the established features of KPLBS [1]. The triad of infantile spasms, profound axial hypotonia, and the specific facial dysmorphism was the key clinical indicator. The normal head circumference in our patient is a slight deviation from the microcephaly often reported, suggesting phenotypic variability and expanding the recognized clinical spectrum [3]. The normal EEG and MRI are of particular interest, as they highlight that KPLBS, being a channelopathy, may not present with structural brain anomalies or interictal epileptiform activity on surface EEG, often leading to initial diagnostic uncertainty [5]. This normal EEG, despite clear clinical seizures, may reflect the limitation of routine scalp EEG in capturing the diffuse or deep cortical hyperexcitability caused by a global ion channel dysfunction.

The identified *KCNJ6* variant is believed to cause a gain-of-function in the *GIRK2* channel [1]. This aberrant function impairs the normal selectivity and kinetics of potassium efflux, preventing effective repolarization of neurons after depolarization. The resultant sustained depolarization lowers the seizure threshold and leads to neuronal hyperexcitability, clinically manifesting as infantile spasms and other seizure types. Furthermore, research suggests abnormalities in glial potassium channels (e.g., *Kir4.1*) also contribute to epileptogenesis by failing to buffer extracellular potassium, exacerbating neuronal depolarization. This highlights the complex interplay in channelopathies and points to a potential future direction for targeted therapeutic research beyond neuronal targets alone.

The management of seizures in ultra-rare channelopathies like KPLBS is inherently empirical, as no clinical trials or consensus guidelines exist. For the infantile spasms observed in this case, first-line agents per general infantile spasm guidelines, such as adrenocorticotropic hormone (ACTH) or vigabatrin, are appropriate considerations. For broader seizure management, broad-spectrum antiseizure medications (ASMs) such as levetiracetam, valproic acid, or topiramate are often used. Lamotrigine may also be considered. Importantly, drugs that potentiate GABAergic inhibition (e.g., barbiturates and benzodiazepines) require caution. Since GIRK2 channels are coupled to GABA-B receptors, enhancing this pathway could theoretically further potentiate the dysfunctional channel's activity, potentially worsening hyperexcitability, though clinical data are scarce. The response to any ASM must be closely monitored.

Given the certainty of severe global developmental delay and high risk of drug-resistant epilepsy, early involvement of a multidisciplinary team is paramount. This team should include a pediatric neurologist, a medical geneticist, a nutritionist (for anticipated feeding difficulties), and palliative care specialists to provide comprehensive supportive care from the time of diagnosis. Evaluation by a medical geneticist is essential for coordinating diagnostic confirmation, interpreting complex genetic results, and providing comprehensive genetic counseling to the family regarding recurrence risks and reproductive options.

The prognosis for individuals with KPLBS is uniformly guarded. Neurodevelopmental outcome is universally poor, with most patients not achieving independent sitting, walking, or speech [1,3]. Seizures are a persistent comorbidity. Feeding difficulties frequently necessitate gastrostomy tube placement. The focus of management shifts definitively to palliative and supportive care, aiming to control seizures, manage feeding difficulties, prevent complications like aspiration pneumonia, and optimize quality of life.

## Conclusions

This case confirms the diagnosis of KPLBS in a Lebanese infant through WES, demonstrating the critical role of genomic testing in solving complex neurodevelopmental presentations. The normal neuroimaging and EEG underscore that KPLBS is primarily a clinical and genetic diagnosis. The management of epilepsy is challenging and requires a tailored, empirical approach, considering both general guidelines for seizure types (e.g., infantile spasms) and the theoretical implications of the underlying channelopathy. The prognosis remains severe, necessitating the early formation of a multidisciplinary care team including neurology, clinical genetics, nutrition, and palliative care to provide comprehensive supportive care. Comprehensive genetic counseling facilitated by a medical geneticist is an indispensable component of care, providing the family with critical information and support.
